# Advance Planning for Technology Use in Dementia Care: Development, Design, and Feasibility of a Novel Self-administered Decision-Making Tool

**DOI:** 10.2196/39335

**Published:** 2022-07-27

**Authors:** Clara Berridge, Natalie R Turner, Liu Liu, Sierramatice W Karras, Amy Chen, Karen Fredriksen-Goldsen, George Demiris

**Affiliations:** 1 School of Social Work University of Washington Seattle, WA United States; 2 College of Education University of Washington Seattle, WA United States; 3 Clinical Informatics Research Group Biobehavioral Nursing and Health Informatics University of Washington Seattle, WA United States; 4 School of Nursing and Perelman School of Medicine University of Pennsylvania Philadelphia, PA United States

**Keywords:** Alzheimer disease, advance care planning, dyadic intervention, technology, remote monitoring, artificial intelligence, older adult, seniors, human-computer interaction, aging, elderly population, digital tool, educational tool, dementia care, ethics, informed consent

## Abstract

**Background:**

Monitoring technologies are used to collect a range of information, such as one’s location out of the home or movement within the home, and transmit that information to caregivers to support aging in place. Their surveilling nature, however, poses ethical dilemmas and can be experienced as intrusive to people living with Alzheimer disease (AD) and AD-related dementias. These challenges are compounded when older adults are not engaged in decision-making about how they are monitored. Dissemination of these technologies is outpacing our understanding of how to communicate their functions, risks, and benefits to families and older adults. To date, there are no tools to help families understand the functions of monitoring technologies or guide them in balancing their perceived need for ongoing surveillance and the older adult’s dignity and wishes.

**Objective:**

We designed, developed, and piloted a communication and education tool in the form of a web application called Let’s Talk Tech to support family decision-making about diverse technologies used in dementia home care. The knowledge base about how to design online interventions for people living with mild dementia is still in development, and dyadic interventions used in dementia care remain rare. We describe the intervention’s motivation and development process, and the feasibility of using this self-administered web application intervention in a pilot sample of people living with mild AD and their family care partners.

**Methods:**

We surveyed 29 mild AD dementia care dyads living together before and after they completed the web application intervention and interviewed each dyad about their experiences with it. We report postintervention measures of feasibility (recruitment, enrollment, and retention) and acceptability (satisfaction, quality, and usability). Descriptive statistics were calculated for survey items, and thematic analysis was used with interview transcripts to illuminate participants’ experiences and recommendations to improve the intervention.

**Results:**

The study enrolled 33 people living with AD and their care partners, and 29 (88%) dyads completed the study (all but one were spousal dyads). Participants were asked to complete 4 technology modules, and all completed them. The majority of participants rated the tool as having the right length (>90%), having the right amount of information (>84%), being very clearly worded (>74%), and presenting information in a balanced way (>90%). Most felt the tool was easy to use and helpful, and would likely recommend it to others.

**Conclusions:**

This study demonstrated that our intervention to educate and facilitate conversation and documentation of preferences is preliminarily feasible and acceptable to mild AD care dyads. Effectively involving older adults in these decisions and informing care partners of their preferences could enable families to avoid conflicts or risks associated with uninformed or disempowered use and to personalize use so both members of the dyad can experience benefits.

## Introduction

### Background

Reducing unnecessary care transitions while enabling aging in place is widely considered a priority in the context of a shortage of human and financial resources for elder care [1-3]. To address this challenge, policy makers, providers, and family caregivers are looking to technological solutions and investing in internet-connected devices that monitor the activity and safety of older adults with Alzheimer disease (AD) and AD-related dementias (ADRD), including technologies that involve artificial intelligence and natural language processing [4-12]. A problem that accompanies this shift is that the dissemination of technologies that passively collect and transmit personal data is outpacing our understanding of how to help families think about and involve older adults in decisions about if and how they want to be monitored. When families are not equipped to make informed decisions about technology use and to match a device to their needs and values, they are unlikely to use or benefit from it [4,13], and ill-informed decisions expose them to the technologies’ risks. 

Helping families navigate the complex technological landscape is a timely goal. Researchers often highlight the core ethical problem of achieving informed consent with an individual living with dementia [14-18]. They have demonstrated the need for tools to support education, awareness, and decision-making about technologies used to support care [16,19-21], including forward-looking consent processes before dementia undermines informed preference formation or expression [22]. This article reports on a novel self-administered intervention, Let’s Talk Tech, to address this problem, which is modeled on advance care planning interventions. The goal is to meaningfully engage people living with mild dementia in planning for the use of technology in their care and to enable understanding of the implications of technology use and communication about it, so families are not left to navigate this complex space alone. 

Let’s Talk Tech supports decision-making about the use of these technologies and will advance the scientific understanding of how to engage people with early stage AD/ADRD in these decisions to enable their personalized use. To evaluate the intervention’s feasibility, usability, and acceptability, we conducted a pilot study with 29 mild AD dementia care dyads. In this paper, we describe the development of the intervention to engage older adults in decisions about technology used in their care, report findings on study endpoints of feasibility and acceptability, and discuss key insights to support successful online intervention development with dementia care dyads.

### Problems This Intervention Targets

The passive collection of location, audio, video, movements, and activities in elder care, and dementia home care in particular, is becoming more common [14,23]. Empirical and ethics research have established that the potential benefits of technologies with remote monitoring functions come with potential risks, and these have not been presented for consumers to understand. The dominant risks and challenges that are innate to passive monitoring are in conflicts among privacy, autonomy, freedom, and safety and risk management [24]. Potential risks include isolation through reduced human interaction and hands-on care, privacy invasion, loss of control, data inaccuracy, and reduced behavioral autonomy [14,16,17,22,25-36]. Research indicates that it is not easy for older adults on their own to appreciate what it will be like to be monitored [37]. Pragmatically, it is wasteful when families invest in technologies that do not ultimately work for them. Preventing this and maximizing the potential benefits require the right balance and respect for boundaries that are specific to each family or care partnership.

### Potential for Dyadic Incongruence, Conflict, and Stress

The use of technologies that collect data, such as visual, activity, location, and audio, in dementia care may contribute to familial stress and conflict due to their surveilling nature. Studies also indicate the potential for incongruent preferences and difficulty navigating these decisions in a way that respects the values held by the older adult who the technology would be used on or with [17,27,30,37-40]. For example, in a dyadic study of Meals on Wheels clients and their primary family support person, adult children and their parents expressed conflicting views about how and when location tracking technology, in-home sensors, and web cameras should be used [27]. Adult children felt that involving their parents in conversations and decisions about whether to use these kinds of technologies would be complicated, underestimating their capacity to understand the technologies, and most felt that they would involve parents minimally [27].

Disagreement about treatment preferences has been associated with caregiver depression [41]. Dyadic strain has been associated with lower quality of life for African American dementia caregivers, and involvement in decision-making and concordance has been associated with quality of life for people living with dementia [42]. Incongruent appraisal of care values may contribute to worse quality of life for both members of the dementia care dyad [43].

Our hope, therefore, is that this communication tool will prepare care partners to make decisions that they feel confident about, support ethical application, and uphold the dignity and rights of people living with dementia. Involvement in planning recognizes the personhood of people living with dementia and the fact that they have preferences that can be expressed [44,45]. Care dyads require support to identify person-centered values in relation to technologies and practical tools to select the options that align with those values as needs change.

There is no tool available to families to facilitate conversation, decisions, or planning for technology used to support elder care. Web-based behavioral interventions for older adults can be made freely available and have been found to be feasible and acceptable [46], yet there are few interventions that support dyadic dementia care partners to plan for care [47]. Dementia care apps have the potential to improve quality of life for people living with dementia and care partners [46], but their development requires consideration of a range of needs [48]. The research on usability and needs with regard to apps to support dementia caregiving or dyads is at an early stage [16,49-51]. The development of Let’s Talk Tech was informed by the limited research on human-computer interaction–related best practices and techniques to support people living with dementia through online interventions [52]. As such, these pilot feasibility findings should help inform future design directions, particularly for web application development. 

## Methods

### The Intervention

The intervention is in the form of a self-administered web application completed by a person with early stage AD (here, “person living with dementia”) together with their primary care partner in an active collaborative process that involves education, discussion questions, and the documentation of the preferences expressed by the person living with dementia. Let’s Talk Tech guides them through a discussion about what technologies they might want and under what conditions they would want them to be used. It requires no trained professional but requires that at least two people sit down together and use an internet-connected device to complete it. 

The intervention’s purpose is to facilitate communication and sharing of preferences similar to what has been developed for decisions about advance care planning, for the benefit of both members of the care dyad. Its components were modeled on elements of established advance care planning tools, including Your Life, Your Choices [53], and PREPARE [54]. Let’s Talk Tech’s flexibility to be retaken, referred to at a later date, and edited is informed by the research conducted by Sudore and Fried, in which decision-making was conceptualized as a dynamic process of communicating values [55]. An aim of the intervention, therefore, is to improve, as potential surrogates, care partners’ knowledge of the technology preferences of the person living with dementia and related values to prepare them to make the best decisions in the future should the person lose capacity to participate [55].

### Development of Let’s Talk Tech

The web application intervention’s content was developed from a 2-study process that generated cross-stakeholder input from key groups who had not previously been engaged in the same knowledge production process. First, in order to identify the technologies that should be included in the tool and information that could help families understand the implications of use, we employed the Delphi approach to achieve consensus from gerontechnology domain experts in the United States and Canada, to identify the salient risks and benefits associated with specific technologies predicted to be commonly used in home dementia care in the near future [14]. Domain experts also ranked these technologies according to those most likely to warrant a conversation with the person living with dementia to ensure acceptable use. We selected 4 technology categories from this list with an eye on the collective variability of data type (location, audio, visual, etc). The technologies featured in the web application are location tracking outside of the home, 4 activity sensors inside the home, web cameras, and artificial companion robots that use artificial intelligence and voice to interact with a person.

In that domain expert study, specific ways to mitigate prominent risks these technologies pose were also identified. These risk mitigation strategies applied to such diverse realms as design, policy, and regulation, and to interpersonal care practices, such as ensuring the ability to pause a device when one wants privacy or to be reminded about what information a given technology is collecting about them [14]. Five of the most commonly endorsed risk mitigation strategy options were incorporated into a survey for 825 people aged 21-92 years, with a mean age of 64 years (SD 13.13 years). The sample included a significant proportion of people who had memory problems or had been seen by doctors about memory concerns (n=201) [56]. The survey assessed the importance participants placed on the 5 actionable risk mitigation strategies for the use of these kinds of technologies in elder care [56]. Findings from the survey confirmed the very high importance and relevance of these 5 options to an older sample of people, including those with and those without reported memory problems [56].

The cumulative findings from the expert study and large survey of older adults were the building blocks of the intervention. The main components of the Let’s Talk Tech web application are 4 featured technology modules (location tracking, in-home sensors, web cameras, and artificial companion robots). The goals of each module are to (1) clearly communicate the function of each technology, (2) clearly communicate the research-based prominent risks and benefits of using each, (3) prompt discussion between dyad members about their feelings, (4) document the preferences of the person living with dementia for use, nonuse, or conditioned use of each technology, and (5) document preferences for the use of alternatives to the featured technologies. Alternatives are offered to ensure that the option to use a given technology is presented as a true choice rather that the only acknowledged option to support care. Participants are presented with clear descriptions of the 4 data-diverse technology categories and prompted to discuss with each other their feelings about them. In order to help the dyad members appreciate what it might be like to use each, the web application presents prominent positive and negative implications for each technology, derived from the expert study [14], and assesses which are of most importance to the person living with dementia. The person living with dementia is then prompted to document their use preference for each, as well as the options that use may be contingent on. After the technology modules are completed, participants are guided through a series of general questions regarding the options that may be important to them, derived from the survey research described above [56]. The web application provides a summary document that summarizes their choices and discussion. It can be accessed and edited any time at a later date.

For accessibility, the web application complies with the criteria of the Web Content Accessibility Guidelines (WCAG) 2.1 at Level AA. The WCAG represent the standard for web accessibility developed by the World Wide Web Consortium. The web application includes an automatic audio option to hear the content read aloud and is screen-reader compatible. To ensure that all aspects of accessibility for those living with mild dementia were considered, the design and content of the tool were shared and discussed with 10 human-computer interaction design experts and clinicians who work with people living with dementia. The clinicians included a neurologist, a neuropsychologist, physicians (eg, geriatricians and palliative care experts), gerontological social workers, and nurses. For example, Let’s Talk Tech’s content was designed to avoid abstractions, and sentences were reviewed for clarity, singular constructs, and shorter length to enable comprehension, in addition to word choice. The verbiage used to introduce the web application on its home page is provided in Textbox 1. 

**An introductory message of Let’s Talk Tech.**

**Why talk about technology now?**

There are many ways to add support to help someone live independently. One is to use technology.

Some technologies collect information about a person to help a family member or caregiver monitor them. The only person who knows if you’re comfortable about any of these technologies is you. Information about what it can be like to use these technologies could help you decide how you feel.

That’s why this can help. Having conversations about technology choices can help you think about what you want or don’t want. Letting people know how you feel can make it easier for them to follow your choices when deciding about using technologies in the future.

The reason to do this together is this can be a shared decision.

### Participants

The inclusion criteria for older adults were as follows: (1) enrollment in the University of Washington Alzheimer’s Disease Research Center (UW ADRC) clinical core or research registry with a diagnosis of mild AD dementia; (2) age 55+ years; (3) English speaking; and (4) having a care partner identified as a primary support person willing to participate in the study. The inclusion criteria for care partners were as follows: (1) co-participant of an ADRC clinical core patient or research registry patient who has been diagnosed with mild AD dementia; (2) identification by a study participant aged 55+ years as someone who is their primary support person; (3) age 18+ years; and (4) English speaking. Between the 2 potential dyad participants, one had to have access to a device (such as a computer, laptop, or tablet) that they could use together, which had an internet connection. Twenty-nine dyads participated in the study. Each individual participant received a Visa gift card for US $150 for their time upon completion of the 3 steps described below. 

### Ethics Approval

The study received approval from the University of Washington Division of Human Subjects (study number: STUDY00014226). Informed consent was obtained from each participant.

### Procedures

Reported in this paper are the feasibility findings for recruitment, enrollment, and retention, and the survey questions and interviews that assess the acceptability of Let’s Talk Tech. Acceptability was assessed at time 2 (T2) after use of the intervention, using 7 survey questions to measure satisfaction, quality, and usability. The specific items are presented with their outcomes in the Results section. T2 surveys were followed immediately by dyadic interviews that probed further about participants’ responses to the acceptability questions. The interviews allowed us to learn about specific components of the web application that worked or did not work well for each dyad, and to identify areas for improvement. The interview portion lasted an average of 33 min (range 15-75 min). Among the dyads, 65% completed these interviews by Zoom video and 35% completed in person. All interviews were audio recorded with permission. 

Procedures for the pilot study as a whole involved the following 3 steps: (1) time 1 (T1) study questionnaire completion individually with the researcher present to support the person living with dementia, if needed; (2) web application completion together as a dyad without the researcher present, and (3) T2 questionnaire completion individually with the researcher present to support the person living with dementia, followed immediately by an interview with the dyad. Questionnaires were administered via REDCap, and printed copies were used for those who requested it. The web application was self-administered, and no researcher was present or assisted dyads with it, apart from showing them how to access it during T1. However, the set of T1 and T2 surveys relied on a researcher to administer the questions to the people living with dementia. Care partners independently completed their surveys in REDCap and a researcher stayed with the person living with dementia to answer clarifying questions as they completed their own surveys in REDCap. In our case, the researcher was a licensed master social worker with clinical experience working with people living with dementia and their care partners.

The study outcome measures, which are not reported here, included 27 questions for care partners and 7 questions for people living with dementia unique to this study to assess knowledge, understanding, and preparedness to make decisions about technology use (primary efficacy outcomes). Both participant groups also completed 2 subscales of the Dyadic Relationship Scale to measure positive dyadic interaction and strain [57] (secondary outcomes) and the Decision-Making Involvement Scale assessing the level of involvement of people living with dementia in daily decisions [58] for descriptive purposes. Care partners were administered the General Anxiety Disorder-7 (GAD-7) [59] to confirm that the intervention would not increase anxiety, and the Stetz Inventory to describe this participant group’s level of involvement with caregiving tasks [60,61].

### Analysis

Analyses for descriptive statistics and frequency counts were performed in R (R Core Team). Frequency counts were used to summarize participant T2 feasibility and acceptability results, and T2 transcribed interviews were coded in Dedoose (Version 9.0.17; SocioCultural Research Consultants, LLC). Two coders used a process of thematic analysis to identify themes regarding participants’ experiences with the web application and suggestions for improvement [62,63]. A codebook was developed based on the interview guide followed by initial coding by a primary coder who developed inductive codes in the process. The new codes were incorporated, and a secondary coder then reviewed the coding decisions and the 2 discussed discrepancies and reached consensus about them [63]. The pair then read the coded excerpts across interviews and identified themes related to outcomes of feasibility and acceptability.

## Results

### Feasibility

#### Recruitment, Enrollment, and Retention

Recruitment was conducted through 2 existing university research volunteer pools who had consented to be contacted about potential participation in other studies. As part of the UW ADRC’s operations, both the clinical core patient participants and their co-participants have annual visits with UW ADRC, and the status of mild AD dementia is reassessed. The ADRC prescreened participants in their research registry group to identify those with mild AD dementia and those with mild AD who also had a co-participant (here, “care partner”) volunteer for our recruitment list. Because the UW ADRC diagnoses the patients and reassesses them annually and because the center has diagnosis and severity information for the participants, there was no further assessment to determine cognitive impairment status. 

Those who were identified by the ADRC as having a diagnosis of mild AD dementia were invited to participate with their care partner by phone or email according to their preferences for a total of 110 people living with dementia invited. Thirty did not respond to the invitation, and we do not know the reasons for their nonparticipation. The reasons for nonparticipation among respondents were as follows: care partners determined that the people living with dementia had dementia too far advanced (n=11), not interested (n=11), not a good time (n=9), and lack of a device or comfort using a computer (n=2). Thirty-three people living with dementia enrolled with their care partners, and 29 (88%) dyads completed the study. Of the 4 dyads who did not complete the study, 1 dropped out before T1 because of difficulties with a recent move to memory care, 2 dropped out during T1 because the standardized survey scales were too difficult for the people living with dementia, and 1 dropped out after T1 because of computer difficulties generally and because the care partner had an overwhelming health change.

Age, gender, race, and ethnicity reported by both people living with dementia and care partners are presented in Table 1. Care partners were mainly spouses, and 1 care partner was an adult daughter. Participants wrote in their gender identity. Among the participants, 38% (11/29) of care partners and 62% (18/29) of people living with dementia were male. The age of care partners ranged from 55 to 83 years (mean 68 years, SD 6.73 years), and the age of people living with dementia ranged from 59 to 82 years (mean 70 years, SD 7.06 years). Only 3 participants did not identify as non-Hispanic white (2 Asian American care partners and 1 African American person living with dementia). Data on Hispanic/Latino ethnicity were missing for 7 care partners. 

**Table 1:** Participant characteristics.

Demographics		Care partner (N=29)	People living with dementia (N=29)
Age (years), mean (SD); range		68 (6.73); 55-83	70 (7.06); 59-82
**Gender, n (%)**			
	Male	11 (38)	18 (62)
	Female	18 (62)	11 (38)
**Race, n (%)**			
	White	27 (93)	28 (97)
	African American	0 (0)	1 (3)
	Asian	2 (7)	0 (0)
Hispanic/Latino ethnicity, n (%)		0 (0)	0 (0)

#### Completion

Our a priori cut point, at which the intervention is considered complete, was if the dyad completed at least three of the four technology modules. Participants were asked to complete all modules, but this was a self-administered intervention in which the researcher was not present while the dyads worked through the web application. As such, we did not expect the high completion rate of 100% for the 4 modules. As a group, participants completed 98.4% of the primary 17 questions asked in the web application (a total of 485 of 493 nonskip logic follow-up questions). Two participants did not answer 1 question each, 1 did not answer 2 questions, and 1 did not answer 4 questions. Only 2 of the dyads reported spreading the web application over 2 sessions. On average, the time between T1 and T2 was 16 days. The average time between web application completion and T2 was 4 days.

### Acceptability: Satisfaction, Quality, and Usability

Satisfaction, quality, and usability were measured with Likert response item questions and follow-up interviews to probe responses. Satisfaction was measured using the following questions answered on 5-item Likert scales: “How helpful was the tool?” (“Extremely unhelpful” to “Extremely helpful”) and “How likely would you be to recommend this tool to others living with dementia or their caregivers?” (“Extremely unlikely” to “Extremely likely”). Quality was assessed with the following questions: “Was the tool balanced?” (“Slanted in favor of using the technology,” “Slanted against using the technology,” and “Balanced”) and “Was there enough information to help you decide about how to answer the questions?” (“Too much information,” “Too little information,” and “Just right”). Questions about ease of use, clarity, and length describe usability as follows: “How easy was it to use this tool?” (“0 [very easy]” to “10 [very hard]”), “Were the descriptions clearly worded?” (“Very clearly,” “Somewhat clearly,” and “Not clearly”), and “Please rate the tool’s length” (“Too long,” “Too short,” and “Just right”). These findings are presented in Table 2. Semistructured dyadic interviews immediately followed this questionnaire to probe these responses and to learn about participants’ experiences with the intervention. We also present interview themes that provide greater insight into survey responses about feasibility and acceptability.

As depicted in Table 2, all care partners answered all satisfaction, quality, and usability questions, and depending on the question, 4 to 5 people living with dementia did not answer because they reported difficulty remembering the web application experience well enough to answer the questions. Both participant groups generally reported that Let’s Talk Tech’s length was just right. Overall, 80% (23/29) of care partners and 68% (17/25) of people living with dementia who answered the question felt that the descriptions were very clearly worded. One care partner reported that the descriptions were not clearly worded. Moreover, 86% (25/29) of care partners and 83% (20/24) of people living with dementia said that the amount of information was just right, while 14% (4/29) of care partners and 13% (3/24) of people living with dementia said there was too little information, with 1 person living with dementia reporting too much information. Some dyads specifically noted the need for more concrete and visual examples, particularly about what an artificial companion robot could do. Some participants suggested ways to enable a deeper dive into the technologies in each module for those who wanted to learn even more, including how to find a device or product on the market. 

**Table 2:** Feasibility measures of satisfaction, quality, and usability for care partners and people living with dementia at time 2.

Question and responses		Care partners (N=29), n (%)		People living with dementia (N=29), n (%)	
		Completed	Missing	Completed	Missing
**Please rate the tool’s length**		29 (100)	0 (0)	24 (83)	5 (17)
	Too long	2 (7)		1 (4)	
	Too short	0 (0)		1 (4)	
	Just right	27 (93)		22 (92)	
**Were the descriptions clearly worded?**		29 (100)	0 (0)	25 (86)	4 (14)
	Not clearly	1 (3)		0 (0)	
	Somewhat clearly	5 (17)		8 (32)	
	Very clearly	23 (80)		17 (68)	
**Was there enough information to help you decide about how to answer the questions?**		29 (100)	0 (0)	24 (83)	5 (17)
	Too much information	0 (0)		1 (4)	
	Too little information	4 (14)		3 (13)	
	Just right	25 (86)		20 (83)	
**Was the tool balanced?**		29 (100)	0 (0)	24 (83)	5 (17)
	Slanted in favor of using the technology	2 (7)		3 (13)	
	Slanted against using the technology	0 (0)		0 (0)	
	Balanced	27 (93)		21 (87)	
**How helpful was the tool?**		29 (100)	0 (0)	24 (83)	5 (17)
	Extremely unhelpful	0 (0)		0 (0)	
	Unhelpful	0 (0)		1 (4)	
	Neutral	2 (7)		3 (13)	
	Helpful	25 (86)		18 (75)	
	Extremely helpful	2 (7)		2 (8)	
**How likely would you be to recommend this tool to others living with dementia or their caregivers?**		29 (100)	0 (0)	25 (86)	4 (14)
	Extremely unlikely	1 (4)		0 (0)	
	Unlikely	0 (0)		1 (4)	
	Neutral	5 (17)		5 (20)	
	Likely	18 (62)		14 (56)	
	Extremely likely	5 (17)		5 (20)	

Participants felt Let’s Talk Tech was balanced, except for 7% (2/29) of care partners and 13% (3/24) of people living with dementia who felt it was slanted in favor of using the technology. None felt it was slanted against use. A couple of participants noted that having an intervention that has a purpose to encourage discussion about technology options causes bias toward technology (eg, “maybe it’s the fact that here’s some offer of technology to help. You know, not that you’re pushing it but it’s there. So it feels like it's an automatic pro for the technology”). Others appreciated the neutralizing features of the tool, specifically, presentation of nontechnology alternative options to support care, as well as both positive and negative aspects of each technology. One care partner explained, “the pros and cons examples were very good and I think those are very important. Because otherwise it can be very leading…I thought you did a good job, because otherwise, if you just list all the pros your brain goes that way.” Another care partner elaborated, “the format’s conducive to being honest with it. It doesn't promote trying to gain anything. It's pretty neutral that way.” 

On a scale of extremely unhelpful to extremely helpful, 86% (25/29) of care partners and 75% (18/24) of people living with dementia rated the intervention as helpful, with 2 in each group rating it as extremely helpful, and 2 care partners and 3 people living with dementia selecting neutral. One person living with dementia rated it as unhelpful. Additionally, 79% (23/29) of care partners and 76% (19/25) of people living with dementia were likely or extremely likely to recommend Let’s Talk Tech, while 1 person living with dementia was unlikely and 1 care partner was extremely unlikely to recommend Let’s Talk Tech. Interviews revealed that most of the individuals who reported that they would be likely to recommend it to others cited the benefit of awareness gained about technological tools that may be helpful and the support with having conversations about them, and some who were not inclined felt it would not be their business to make such a recommendation.  

Roughly half of the dyads reported some discomfort in completing Let’s Talk Tech, noting that thinking about the need for technologies is scary or unsettling, that any disagreement is hard, or that it can bring up worries about being a burden for people living with dementia. However, all stated that it was worth the discomfort. For example, a dyad explaining that it makes people living with dementia very sad to talk about advance planning for care support, discussed why that was worthwhile as follows:

And by having these conversations, makes it easier for both of us, because then we're not guessing.

Care partner

It's true and, and the more we're able to talk about it, the more comfortable it is, that okay, this is just how things are now and it's okay.

Person living with dementia

And we can joke about it.

Care partner

When asked directly if it was worth the sadness people living with dementia felt, this person living with dementia responded, “Oh absolutely yes. Yes, because it’s something to get through. And the only way to get to the other side is to talk about it and yeah absolutely.”

**Figure 1:**
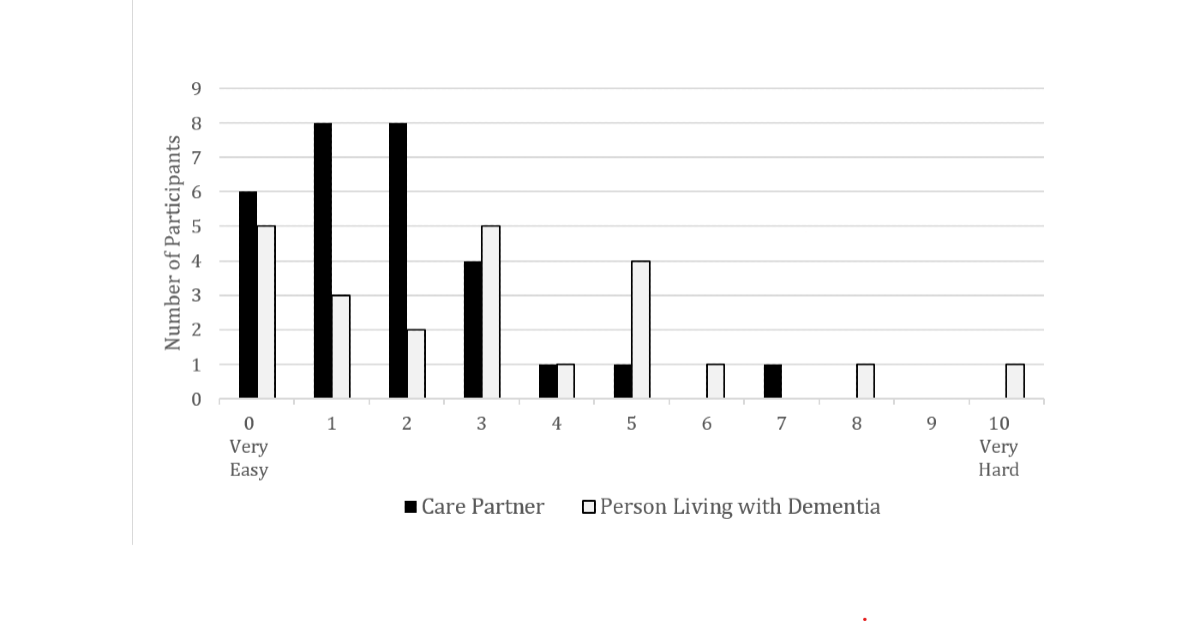
Web application tool ease of use. Frequency responses to “How easy was it to use this tool?”.

Participants were asked how easy it was to use Let’s Talk Tech. Six of the people living with dementia did not remember it well enough to answer. Figure 1 presents a visual comparison of the 2 participant group ratings of ease of use. It was harder for people living with dementia as compared with care partners, though both groups primarily reported it as somewhat easy. Four participants (3 people living with dementia and 1 care partner) rated the tool difficult to use (score of 6 or greater). When asked about this rating, 1 person living with dementia said it was because this was the first time she was thinking about this topic and she was trying to wrap her head around the technology. Another had trouble remembering that he had used the web application and was in pain during the interview, so he did not expand on the reason for his rating. The dyad that rated the tool difficult to use reported difficulty in relating scenarios specified in the tool to their own lives and felt that they were too broad.

Interviews confirmed the survey findings that the intervention is most usable and useful during early/mild stages of dementia when using the tool is not too onerous for people living with dementia, and it is easily navigated with questions well understood. Some people living with dementia felt that they were not at a stage of their disease that warranted the use of the featured technologies and thus had difficulty relating to the questions about their preferences for them as they felt they were not needed. Not all struggled with this, but participants from 12 dyads (11 care partners and 4 people living with dementia) recommended including more scenarios to enable people to imagine times in the future when their responses or preferences may change. 

Caregivers also indicated that they felt that the disease stage would impact the person’s answers to the questions posed in the web application. While recruitment was conducted with those identified by the ADRC as in a mild stage of AD, we did not conduct additional tests to confirm the current status. Two care partners explained in their T2 interviews that they believed the patients were in the middle stages of the disease. One care partner explained why she thought the ideal time to use Let’s Talk Tech would be at an early stage:

I feel like we’re like moderate like in the middle stages like right in the middle of the middle stage, and so I almost think that in the early stages of, of Alzheimer’s or like right in the beginning of the moderate stage. I mean he can still answer the questions now. It just takes a lot of like rephrasing.

This person felt that had the patient still been at an early stage, he would have been able to answer with better judgment, a more accurate understanding of his own condition, and greater consideration of the demands on her as a care partner, and would have felt less worried about being judged (amplified via a camera, for example) than he was at this moderate stage. Care partners who doubted their partners’ comprehension often also doubted the validity of their responses, making the intervention less helpful as a planning tool for those participants whose AD had advanced beyond the early stage. 

## Discussion

### Principal Findings

The findings of this study represent promising feasibility data for a self-administered web application intervention designed for people living with mild AD/ADRD and a care partner. Participants were able to navigate through the entirety of Let’s Talk Tech and perceived value in the discussions it facilitated despite some discomfort with advance care planning. The high completion of all aspects of the web application was particularly encouraging because we anticipated that working through all modules in one sitting could be a challenge for people living with dementia. Only 2 dyads reported splitting their session with the application into 2 sittings. Participants may have completed all web application forms because they were asked to as part of the introduction of the study, and we should thus not expect such a high rate of completion outside of a study context. It is likely that in a real-world nonstudy context, participants may only complete those modules that seem of particular interest or relevance to them. Still, the successful completion of the Let’s Talk Tech intervention that dyads achieved, primarily in 1 sitting, indicates that the intervention is not too strenuous for care partners or people living with mild AD and is well targeted for this group.

Having difficult conversations was not reportedly a problem for our sample. The interviews described that it was uncomfortable for some, but not so uncomfortable that it outweighed the benefits of having these conversations. This is an important element of feasibility and a promising finding that people may accept this intervention as an opportunity to have conversations they feel are important, though difficult to facilitate on one’s own without such a tool. 

### Limitations and Implications for Future Work

While the feasibility and acceptability ratings were all high, some limitations of the intervention were illuminated by participants through interviews. First, while our findings clearly indicated that Let’s Talk Tech is very well targeted to people living with early stages of AD, a difficulty for people living with mild dementia is that they may not yet feel that there is need for the technologies featured in the intervention. Sometimes there may be disagreement with a care partner about this if they assess their condition differently. Adding future-oriented scenarios would be a clear response to this issue, and participants suggested this directly; however, research also shows that people have a very difficult time projecting themselves into future scenarios with accuracy [64]. Another complication of this potential approach is that a common symptom of AD/ADRD is difficulty with abstract thinking, which makes advance planning and imagining oneself in future or imaginary scenarios challenging [65]. Still, the need to enable the expression of preferences for future scenarios in addition to current use was a strong interview theme, indicating that more research is needed on how to enable this in a way that meets the needs of both members of the care dyad. 

Second, 2 care partners described their partners as being at mid-stage and no longer at the early stages of dementia, and those individuals had difficulty with comprehension. These participants still completed Let’s Talk Tech, but care partners reported more work to navigate it to the point where it could become too onerous and where the responses of people living with dementia could be deemed less reliable by care partners. This underscores our finding that this self-administered web application is well suited for people who have not yet progressed to moderate stages of AD dementia. This also suggests that more research is needed to find ways to engage dyads at moderate stages, such as additional support to answer questions.

Third, the finding that the intervention’s bias toward technology use was mitigated by not naming specific products or devices was not consistent with the finding that dyads would have considered photographs useful for comprehension, and many care partners desired links and next steps to find devices for purchase. While they often had enough information to form preferences, some people living with dementia and care partners reported that they lacked clarity about the scope of what an artificial companion robot could do. This is unsurprising given the relatively low levels of algorithmic awareness [21] and lower familiarity with a more recently developed technology, such as artificial companion robots, relative to location tracking and other featured technologies. It indicates that focus is needed on how to describe the capabilities of such a device, and possibly others using artificial intelligence and natural language processing specifically, in ways that are more likely to be clear to people living with mild dementia and their care partners. Because algorithmic awareness is also associated with categories of socioeconomic status and may be associated with race and ethnicity, it will be important in future studies to collect education, income, and wealth data, and to ensure racial diversity in study samples. A limitation of this pilot study is that we were unable to examine potential associations by these categories.

Fourth, obtaining feedback on the web application days after completion from people living with dementia was sometimes challenging owing to their difficulty with short-term memory. Because it is critical that researchers understand this participant group’s experience with the intervention, creative solutions, such as soliciting real-time feedback during or immediately after use of the intervention, are required.

Finally, having a dementia-trained researcher to administer the survey portions of this study was helpful to guide people living with dementia through a long set of surveys, clarify interview questions, and be sensitive to signals that it was time to stop or pause. Because of this researcher’s clinical experience, we were able to closely observe the points at which participants living with dementia reached their limit with regard to answering research questions. We found that speaking beyond 30 to 35 minutes was sometimes difficult for people living with dementia, at which point answering questions started to become cognitively taxing. They often reported a lack of attention after that point or feeling tired. This observation may be informative for other intervention studies involving people living with mild AD. 

### Conclusion

The use of in-home monitoring technologies to predict health problems and support aging in place is growing faster than our understanding of how to help families make decisions about how and when to use them. Our pilot study findings demonstrate strong preliminary feasibility and acceptability of the Let’s talk Tech intervention for promoting informed shared decision-making about technologies used in dementia care. Successful recruitment, enrollment, and retention, and 100% completion of the web application intervention demonstrate strong feasibility. Good ratings were given for the satisfaction, quality, and usability measures of acceptability. Our findings also revealed useful considerations for other self-administered web application interventions for people living with mild AD and care partners, including optimal exit interview time and the potential need for immediate feedback processes upon intervention completion. Most importantly, this pilot study demonstrated that a self-administered dyadic intervention in the form of a web application can be successfully independently completed in 1 sitting by mild AD care dyads. This research advances the scientific understanding of how to engage people living with dementia in decisions while helping families navigate a complex technology landscape. 

## Abbreviations

AD: Alzheimer disease
ADRC: Alzheimer’s Disease Research Center
ADRD: Alzheimer disease–related dementias
UW: University of Washington
WCAG: Web Content Accessibility Guidelines

## Data Availability

The data sets generated during this study are not publicly available due to the limitations on data reuse as part of the consenting procedures.
